# Preparation and Characterization of Cellulose Nanocrystals from the Bio-ethanol Residuals

**DOI:** 10.3390/nano7030051

**Published:** 2017-02-23

**Authors:** Lanxing Du, Jinwu Wang, Yang Zhang, Chusheng Qi, Michael P. Wolcott, Zhiming Yu

**Affiliations:** 1 College of Material Science and Technology, Beijing Forestry University, Beijing 100083, China; dulanxing@bjfu.edu.cn (L.D.); bjfuzhangyang@bjfu.edu.cn (Y.Z.); qichusheng@bjfu.edu.cn (C.Q.); 2 Composite Materials and Engineering Center, Washington State University, Pullman, WA 99163, USA; jinwuwang@fs.fed.us; 3 Forest Products Laboratory, Forest Service, Department of Agriculture, Orono, ME 04469, USA

**Keywords:** bio-ethanol residuals, cellulose nanocrystal, aspect ratio, particle size, thermal stability, crystallinity, z potential

## Abstract

This study was to explore the conversion of low-cost bio-residuals into high value-added cellulose nanocrystals. Two enzymatic hydrolyzed residuals (i.e., HR_MMW_ and HR_SPW_) were collected from two different bio-ethanol producing processes—hydrolyzing medium-milled wood (MMW) and hydrolyzing acid sulfite pretreated wood (SPW), respectively. The results showed that both residuals contained over 20 wt % glucan with a crystallinity of about 30%, confirming the existence of cellulose in a well-organized structure in two bio-residuals. The cellulose nanocrystals (CNCs) were successfully extracted by first bleaching the hydrolyzed residuals to remove lignin and then hydrolyzing them with sulfuric acid. The resulting CNCs displayed the flow birefringence under two crossed polarizers. Compared with CNCs from microfibrillated cellulose (CNC_MCC_), HR_MMW_ and its resulted CNC present the smallest particle size and aspect ratio. CNC_MCC_ had the larger particle size, aspect ratio, and higher z-potential value, CNC_SPW_ presented a similar morphology to CNC_MCC_, and had the largest aspect ratio. The CNC_MCC_ enhanced its high crystallinity to 85.5%. However, CNC_MMW_ and CNC_SPW_ had a better thermal stability and higher activation energy as well as onset temperature and maximum decomposition temperature. As a result, the CNCs from bio-ethanol residuals are valued and promising cellulose nanoparticle resources.

## 1. Introduction

The advanced bio-ethanol production from lignocellulosic biomass is emerging in response to the depletion of non-renewable petroleum and increasingly environmental concerns regarding the greenhouse gas emissions of using petroleum-based products [1]. It has been reported that advanced bio-ethanol has the potential to cut 86% of greenhouse gases [2]. However, one of the key technological barriers for bio-ethanol production is the low-efficient conversion of biomass into liquid fuels, 32%–35% [3,4,5], which imply that a considerable amount of recalcitrant cellulose, hemicellulose, and lignin are available in the waste streams. In order to minimize the waste of bioconversion and maximize the environmental benefits, the concept of the comprehensive utilization of lignocellulosic biomass has been developed to co-produce bio-ethanol, biochemicals, and biobased products through reorganizing the commercial supply chain. Enzymatic hydrolysis is the first conversion process for bio-ethanol production, the sugar yields from the cellulose and hemicellulose in biomass or biomass residuals were very low, due to the hemicellulose barrier and structural integrity of plant cell walls. They could increase to over 90% after various physical/chemical pretreatments [6]. Enzymes digest the cellulose with loose structure, leaving well organized crystalline cellulose in hydrolyzed residuals whose crystallinity reaches over 74% [7]. Therefore, it is assumed that an appreciable amount of cellulose can be harvested from an industrial-scale bio-ethanol production. The obtained cellulose is typically in the form of powder or granulates because of an upstream grinding step of biomass preparation before conversion. Cellulose powder is of commercial importance in paper making, textiles, drilling, food, chemical engineering, and architecture [8]. In recent years, new research on cellulose nanocrystals (CNCs) obtained by only chemical or physical processing from cellulose has become attractive and promising. A handful investigations have been conducted to extract and utilize the cellulose nanocrystals (CNCs) from hydrolyzed residuals [7,9,10].

Cellulose nanomaterials are high value-added products obtained by isolating cellulose crystallites or microfibrils in the plant cell walls through various processes. Cellulose nanomaterials vary from cellulose nanofibrils prepared by sonication and homogenization to CNCs, a needle-like form of cellulose with an aspect ratio of 10–13 obtained by acid hydrolysis. Microfibrils act as the reinforcement and framework in cell walls [11]. Each individual microfibril has strong physical properties with a tensile strength, bending strength, and Young’s modulus of 2 GPa, 370 GPa, and 138 GPa, respectively [12]. CNCs have been investigated as reinforcement to be added into the polymer matrix like polylactic acid (PLA), Poly(3-Hydroxybutyrate-*co*-3-Hydroxyvalerate) (PHBV), polyethylene (PE), to enhance their mechanical properties [13,14,15]. Functional hydroxyl groups on the surface of CNCs have a strong affinity to themselves and the hydroxyl containing materials and are highly reactive with water [16]. The affinity and hydrophilicity makes CNCs aggregate and distribute unequally, but render them a good absorbent material or matrix [17]. The CNC films had an excellent optical transmission, gas barrier property, and low coefficient of thermal expansion as well as being biodegradable and non-toxic, which has potential in food packaging as well [9,18]. These versatile properties mean cellulose nanocrystals should be studied in widely applications, like cosmetics, pharmaceutical, food packaging, drilling, polymer composites [19,20]. Hence, it is important to find a viable sources and means to prepare them.

The available cellulose resources are mainly from wood and on a lab-scale come from bast or stem fibers, leaf fibers, seed-hair fibers, core, pith or stick fibers, and other resources [21]. Their sizes and particular properties depend on the fibers themselves and diverse processes. In order to enlarge the cellulose resource, the aim of this study was to isolate and characterize the CNCs from two bio-ethanol conversion residuals using the acid hydrolysis coupled with high-intensity ultrasound, and compare them with CNCs from commercial microcrystalline cellulose (MCC). It investigated the technical feasibility of using a novel feedstock from biofuel productions to prepare cellulose and CNCs, even as a replacement for commercial cellulose resources. Making versatile CNCs out of hydrolyzed residuals will bring additional revenue to biorefineries, enhancing bio-ethanol production’s competitive edge.

## 2. Results and Discussion

### 2.1. Chemical Analysis

The chemical compositions of the hydrolyzed residuals and MCC (Table 1) indicate that lignin was the main component in the hydrolyzed residuals. The HR_MMW_ and HR_SPW_ contained 57.74 wt % and 73 wt %, respectively. Glucan content in the hydrolyzed residuals implied that about 60–80 wt % of cellulose was hydrolyzed and 20–40 wt % of recalcitrant cellulose was kept in the residuals. It indicated that there was a relatively considerable amount of cellulose available to be transformed into cellulose or potentially into CNCs. The commercial MCC made with an industrial process contained over 80 wt % cellulose and a little soluble lignin was detectable. The content of glucan was a relatively lower value, probably due to the over-acidic hydrolysis when carrying out the chemical analysis, the glucan was hydrolyzed to the acetylpropionic acid and formic acid.

**Figure 1:**
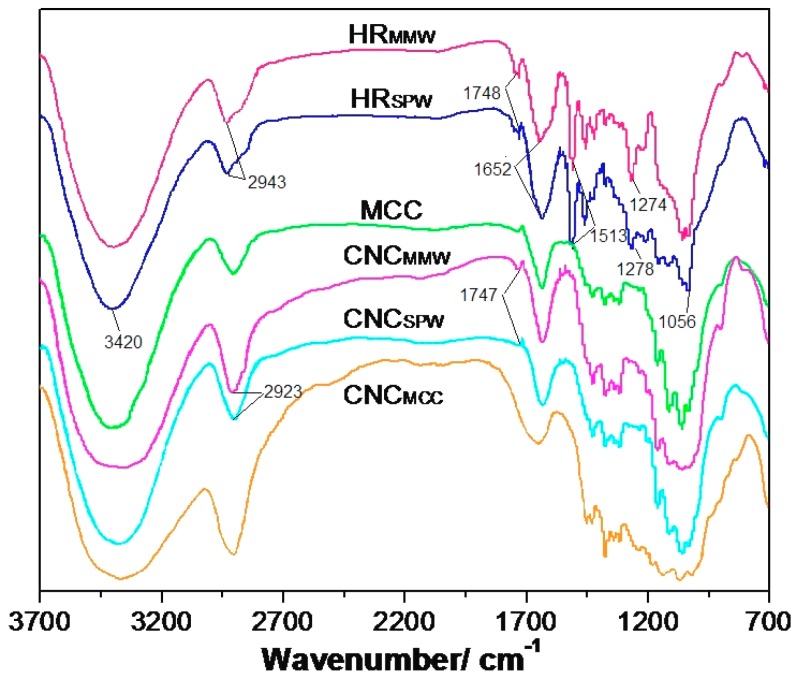
Fourier transform infrared spectroscopy (FTIR) spectra of hydrolyzed residues, MCC, and their cellulose nanocrystals (CNCs).

Fourier transform infrared spectroscopy (FTIR) spectra of the HRs, MCC, and their CNCs were shown in Figure 1. The absorption peaks around 3420, 2943, 1652, and 1056 cm^−1^ contributed to the O–H, C–H stretching, C–C bending, and C–O stretching vibrations on cellulose, respectively [22]. The feature peaks between 1000 and 1170 cm^−1^ were due to the weak C–O stretching vibration [23]. For hydrolyzed residual curves, a carbonyl bond and stretching vibration existed in acetyl group and ester groups from lignin or hemicellulose gave rise to a two absorption features of 1700–1750 cm^−1^, and 1510–1520 cm^−1^ [24]. Both of them disappeared or weakened after bleaching and sulfuric acid hydrolysis. A weak absorption around 1274 cm^−1^ belonging to the C–O stretching of the aryl group in lignin had a similar situation as well. It indicated that the lignin and hemicellulose were removed during the CNC preparation processes. A weak absorption peak at 1747 cm^−1^ in CNC_MMW_ and CNC_SPW_ curves might be due to the partial oxidation by sodium chlorite during the delignification, which introduced a few carboxylic acid groups on the surface of CNCs [25].

### 2.2. Morphology

**Figure 2:**
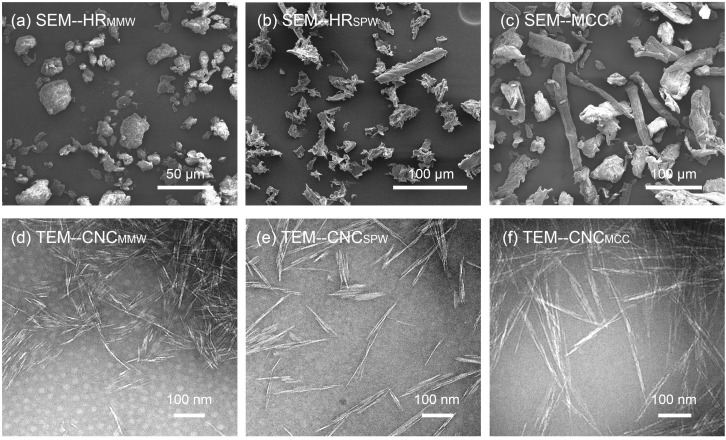
Scanning electron microscope (SEM) and transmittance electron microscope (TEM) images of HR_MMW_, HR_SPW_, MCC, and their CNCs. (**a**) hydrolyzed residues from midium milled wood, HR_MMW_; (**b**) hydrolyzed residues from sulfite pretreated wood, HR_SPW_; (**c**) microcrystalline cellulose, MCC; (**d**) cellulose nanocrystals from HR_MMW_, CNC_MMW_; (**e**) cellulose nanocrystals from HR_SPW_, CNC_SPW_; (**f**) cellulose nanocrystals from MCC, CNC_MCC_. (**a**), (**b**), (**c**) were SEM images and (**d**), (**e**), (**f**) were TEM images

The morphology of the HR_MMW_, HR_SPW_, MCC, and their CNCs are shown in Figure 2. The intensive mechanical forces under the media milling cut the wood fibers in length, destroying their crystalline region and increasing the percentage of amorphous cellulose. The strong friction and shear force reduces the particles size and creates more amorphous regions along with the axis of cellulose chains. Enzymes are prone to digest amorphous regions and the ends of cellulose chains, resulting in the HR_MMW_ from the milled wood becoming almost spherical particles with the average particle size in two dimensions of 13 × 7 μm^2^ (Figure 2a) in contrast with needle-like HR_SPW_ with 34 × 9 μm^2^ particle size (Table 2).

The sulfite pretreatment removed the hemicellulose barrier which was coated on the cellulose, but not cut, furthering the enzymatic hydrolysis, and exposing recalcitrant cellulose. Varied from those two bio-ethanol residuals, the commercial MCC was from cotton with a large particle size of 74 × 20 μm^2^ (Table 2).

**Figure 3:**
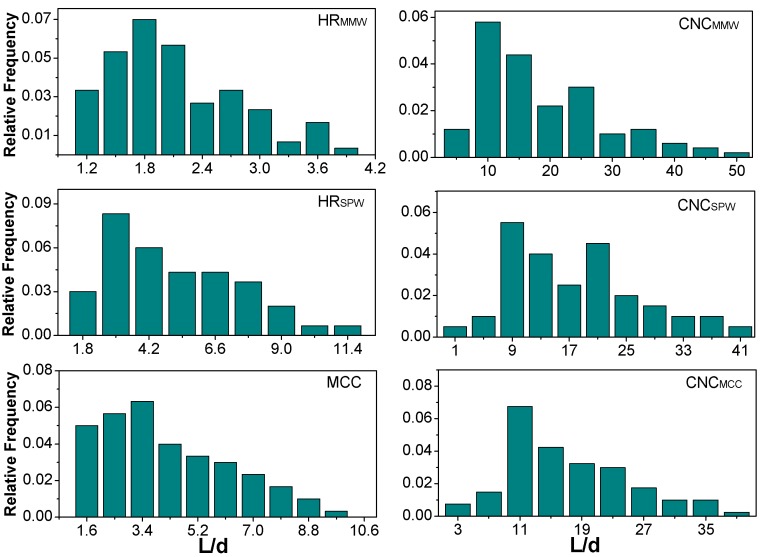
Aspect ratio distributions of HR_MMW_, HR_SPW_, MCC, and their CNCs.

The hydrolyzed residuals after purification and sulfuric acid hydrolysis, and MCC after the same sulfuric hydrolysis isolated CNCs under the width of 100 nm successfully. TEM images of CNCs are shown in Figure 2d–f. CNC_MMW_ had a spindly-like structure and CNC_SPW_ and CNC_MCC_ presented a needle-like structure, as expected. The average length of CNC_MMW_s were 202 nm, making them smaller than those of CNC_SPW_ and CNC_MCC_ at 268 nm and 270 nm, respectively. CNC_SPW_ and CNC_MCC_ exhibited a similar average particle size of length and width, with different aspect ratio distributions (Figure 3). This implied that the pretreatment of medium milling not only affected the yield of sugars and hydrolyzed residuals, but also had an influence on the morphology of final CNCs. The sulfate pretreatment guaranteed the integrity of cellulose. The TEM images showed that CNC_SPW_ and CNC_MCC_ were more individualized compared to CNC_MMW_. One possibility was that a little residual lignin retarding in CNC_MMW_ was sticking CNCs together. As the lignin acted as an adhesive coateing on the surface of CNCs, these sticky CNCs were barely separated in water by simple sonication. The other reason was that the CNC_SPW_ and CNC_MCC_ had a high surface charge. The charged CNCs had the same polarity that was mutually exclusive of uniform dispersion, it was proven in this research and Herrera’s work (Table 3) [9].

**Figure 4:**
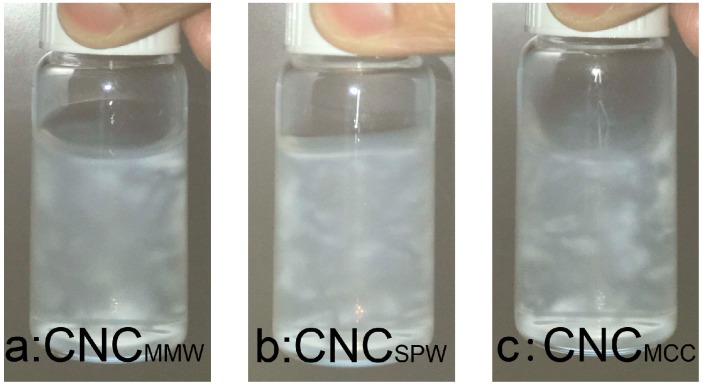
Flow birefringence of cellulose nanocrystal suspensions between two cross polarizers (**a**) CNC_MM__W_, (**b**) CNC_SPW_, and (**c**) CNC_MCC_.

Figure 4 shows intuitive pictures of flow birefringence of CNC suspensions through crossed polarizers. Both CNC_MMW_ and CNC_SPW_ were extracted from these two bio-ethanol residuals. CNC_MCC_ suspension displayed a stronger birefringence due to its well-organized crystal structure with large particle size, other than CNC_MMW_ containing some agglomerate crystals in water.

The histograms of aspect ratios (length to diameter, L/d) as shown in Figure 3 were based respectively on 100 individual particles of HR_MMW_, HR_SPW_, MCC, and their CNCs by analyzing the SEM/TEM images with ImageJ software (National Institute of Health) [26]. The L/d distribution of HR_MMW_ was in a narrow range of 1–3.9. The L/d of HR_SPW_ and MCC showed a broader distribution with a skew towards low aspect ratio values. Their average L/d of HR_MMW_, HR_SPW_, MCC were 2.17 ± 0.91, 5.21 ± 3.45, and 4.20 ± 2.33, respectively. These were corresponding with the SEM images that HR_MMW_ showed a quasi-circular shape, HR_SPW_ and MCC were rod-like shapes. After the sulfuric acid hydrolysis, the average L/ds of CNCs (MMW, SPW, MCC) were 16.0 ± 9.0, 20.8 ± 12.8, and 17.7 ± 6.7, respectively. CNC_SPW_ and CNC_MCC_ had a similar L/d distribution, except the range of 20–23. Because a considerable amount of larger CNC_SPW_ existed, leading to a high average L/d value. In fact, all the CNCs had presented an increased L/d compared with the raw hydrolyzed residuals and MCC, the results even showed that the variation of L/d and its distribution existed in original materials, the sulfuric acid hydrolysis was able to reshape the hydrolyzed residuals and MCC to a similar L/d distribution.

### 2.3. Crystallinity

**Figure 5:**
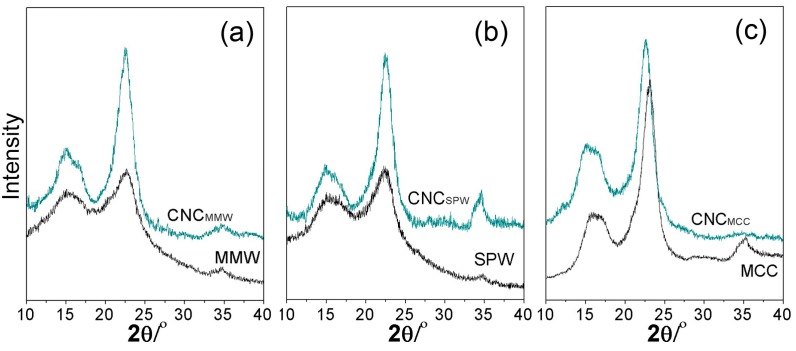
X-ray powder diffractograms of the hydrolyzed residuals (HRs), MCC, and the CNCs. (**a**) MMW and its resulted CNC_MMW_; (**b**) SPW and its resulted CNC_SPW_; (**c**) MCC and its resulted CNC_MCC_.

The crystallinity index (CrI) for CNCs was determined by the following Equation (1) [11,27]:

Cr(%) = (*I*_Max_ − *I*_Am_) / *I*_Max_ × 100 (1)

where *I*_Max_ was the maximum intensity of the diffraction peak, and *I*_Am_ was the intensity of diffraction attributed to amorphous cellulose. The XRD patterns of hydrolyzed residuals, MCC, and their resultant CNCs are shown in Figure 5. Those three CNCs presented four major diffraction peaks around 15.1°, 16.6°, 22.8°, and 34.5°, referring to the cellulose I crystallographic planes 110, 110, 200, and 4, respectively [25]. They were matched with the peaks of hydrolyzed residuals and MCC, without any changes of positions, implying that the sulfuric acid hydrolysis did not change the crystalline structure of raw materials. The comparative crystallinities are listed in Table 3. HR_MMW_ and HR_SPW_ had low crystallinity values and a large amount of cellulose, which mainly provided the organized structure regions, had been enzymatic hydrolyzed. HR_MMW_ contained the lignin and partial of hemicellulose and cellulose. HR_SPW_ had the lignin and recalcitrant cellulose since the sulfite pretreatment removed the hemicellulose with totally arabinan, glactan, and mainly mannan/xylan, shown in Table 1. The HR_MMW_ presented a lower crystallinity due to the intensive force that damaged the organized structure, promoting the enzymatic hydrolysis and then decreasing the percentage of cellulose in hydrolyzed residuals. After the purification and sulfuric acid hydrolysis, the crystallinity of CNC_MMW_ and CNC_SPW_ were almost the same, 73.7% and 74.0%, respectively. It indicated that the hemicellulose, lignin, and the part of amorphous cellulose were removed. Both were lower than that of CNC_MCC_, which was further improved by hydrolysis. It implied that the complicated procedure histories had the possibility of reducing their crystallinity during preparation. However, they still kept a high value of crystallinity.

### 2.4. Zeta Potential

The zeta potential is a valued parameter to evaluate the stability and dispersion of CNCs in aqueous solution. It is calculated using Henry’s equation by determining the electrophoretic mobility by detecting the velocity of the particles using laser Doppler velocimetry [28]. Then zeta potential is calculated as follows:

*U_E_* = 2ε*zf* (*K*a) / 3η (2)

where *U_E_* is the electrophoretic mobility, ε is the dielectric constant, z is the zeta potential, *f*(*K*a) is Henry’s function [28,29], and η is the viscosity. As the cellulose nanoparticles were suspended in aqueous solutions, Smoluchowski approximation was set as the value of 1.5 [30]. The CNC suspensions presented a negative zeta potential (Table 3) attributable to deprotonation of carboxylic acid (only in CNC_MMW_ and CNC_SPW_) and sulfate half esters in neutral water into sulfate anions, providing the repulsion force between the individual CNCs and making them disperse uniformly and stably in aqueous media [31]. Carboxylic acid groups were due to the oxidization of cellulose by sodium chlorite. Sulfate half esters were resulting from the esterification reaction with sulfate anions during sulfuric acid hydrolysis [31,32]. CNC_MCC_ showed the highest negative value of −66.4 mV, and CNC_SPW_ had a higher zeta potential value than CNC_MMW_, implying that more sulfate anions were on the surface of CNC_MCC_. These differences also might be contributed to their different processing histories as CNC_MMW_ had been subjected to lesser chemical treatments.

### 2.5. Thermal Stability

**Figure 6:**
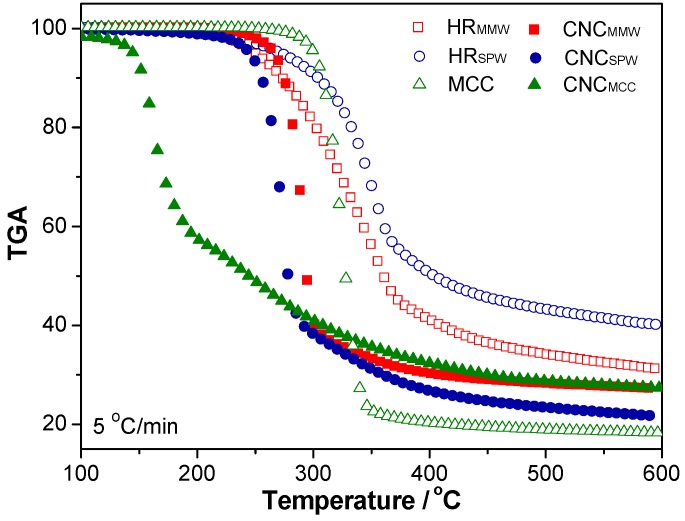
Thermogravimetric curves of the hydrolyzed residuals, MCC and the resulted CNCs.

The TGA curves and onset and peak degradation temperatures (*T*_onset_, *T*_P_) as well as activation energies (*E*_a_) of all materials are shown in Figure 6 and listed in Table 3. *T*_onset_ and *E*_a_ were calculated by the method described in our previous work [33]. Simply stated, the *T*_onset_ and *T*_P_ was described as the beginning of degradation and the temperature corresponding with maximum degradable speed calibrated to the heating rate of 0 °C/min. *T*_P_ was typically designated as the degradation temperature of cellulose. *E*_a_ was calculated by using Kissinger Equation (3):

In(β / T_p_^2^) = In(AR / *E*_a_) + (1 / *T*_p_)(−*E*_a_ / R) (3)

where β was heating rate, *T*_P_ was peak temperature; A was pre-exponential factor; R was the gas constant. −*E*_a_/R was obtained by plotting ln(β/*T*_P_^2^) against 1/*T*_P_ for a series of experiments at different heating rates.

As shown in Table 3, MCC exhibited a high *T*_onset_ value, and a relatively low *T*_P_ value, due to the removal of noncellulosic materials (lignin and hemicellulose) and the usage of chemicals. The chemicals might damage the structure of cellulose during purification that caused the reduction of *T*_P_ value. The acid sulfite pretreatment was used to remove the hemicellulose and helped to digest the cellulose, resulting in HR_SPW_ presenting similar thermal parameters to MCCs [34]. The physical pretreated HR_MMW_, therefore, presented reversed results. *E*_a_ of those three raw materials depending on *T*_P_ showed a similar trend. After the purification, an increased *T*_onset_ of CNC_MMW_ was observed with comparison to HR_MMW_, due to the remaining of stable structure and the removal of the less stable fraction. *T*_P_ values of all CNCs decreased as the concentrated sulfuric acid hydrolysis introduced the sulfate groups on the surface that induced the degradation of CNCs at a relatively low temperature and reduction of thermal stability. The *E*_a_ values of CNC_SPW_ increased slightly. The reason might be the calculation of *E*_a_ used the *T*_P_ value with a varied heating rate. The final *E*_a_ depended on the slope of plotting ln(β/*T*_P_^2^) against 1/*T*_P_. The *T*_P_ values of CNC_SPW_ from 35 °C/min to 5 °C/min decreased slightly and thereby presented a relatively high *E*_a_ value, implying that the heating rate had a considerable influence on the CNC_MMW_ sample and the CNC_SPW_ retained the well-organized cellulose well. The small particle size and the low degree of polymerization might lead to a decrease of *E*_a_, the *E*_a_ of CNC_MMW_ was a little lower than that of CNC_SPW_. The *T*_P_ and *E*_a_ of CNC_MCC_ were the lowest, partially due to its higher degree of the sulfate half esterification during sulfuric acid hydrolysis as indicated by its higher negative zeta potential (Table 3), leading to CNC_MCC_ with a two-step degradation process [7]. A portion of carboxylated CNC_MMW_ and CNC_SPW_ had a better thermal stability than sulfate-decorated cellulose [35]. The carboxyl groups occupied the surface of CNCs that might decreased the opportunity of sulfate half esters decorated on their surfaces (carboxyl groups were proven in FITR). The removal of sulfate groups on cellulose required less energy. They degraded during the first low degradation process, further facilitating the second degradation of cellulose by removing parts of the hydroxyl groups [36].

## 3. Experimental

### 3.1. Raw Materials

Two enzymatic hydrolyzed residuals, from medium-milled wood with 40 min milling time (HR_MMW_) and from acid sulfite pretreated wood (HR_SPW_), were frozen at −20 °C until use. The samples were rinsed by deionized water to remove the remaining sugars, enzymes, and buffers and then freeze-dried before use. Microfibrillated cellulose (MCC) (Fisher scientific, Waltham, MA, USA) was used for control samples.

### 3.2. Cellulose Extraction from Hydrolyzed Residuals

Hydrolyzed residuals (10 g) were mixed with 500 mL deionized water by stirring, they were delignified by adding sodium chlorite (6 g) and acetic acid (5 mL) per hour at 75 °C for 6 h. 2% sodium hydroxide solution (300 mL) was used to purify cellulose by stirring for a 2-h reaction at 90 °C after filtering the delignified holocellulose. These two processes were repeated to guarantee obtaininment of pure cellulose. Both were filtered by a sintered glass filter and washed with deionized water, then freeze-dried and weighed.

### 3.3. Cellulose Nanocrystals Preparation

Freeze-dried cellulose was mixed with 64 wt % H_2_SO_4_ at 1 g cellulose per 9.8 mL acid with strong mechanical stirring at 44 °C for 30 min. The suspension was then diluted 10 times with deionized water and was cooled down in a fridge to stop the reaction. The cool suspension was subject to centrifugation (Sorvall, 5000 rpm for 5 min per time). Supernatant was removed and the solid content was diluted again. The centrifugation steps were repeated until the supernatant became turbid, the turbid supernatant was sonicated for 2 h, and dialyzed against deionized water for a week to remove soluble species.

### 3.4. Characterization

#### 3.4.1. Chemical Analysis

Carbohydrate and lignin contents in the hydrolyzed residuals, cellulose and CNCs were determined by the acid hydrolysis method according to the National Renewable Energy Laboratory’s Laboratory Analytical Procedure [37]. The sample (300.0 mg) was mixed with 3.00 mL of 72 wt % sulfuric acid, and then placed in a water bath at 30 °C for 60 min by stirring every 5–10 min. Then mixture was diluted to a 4% concentration by adding 84.00 mL deionized water before sealed in a pressure tube. A set of reagent sugars including glucose, xylose, galactose, arabinose, and mannose were chosen to most closely resemble the concentrations of sugars in the test samples as sugar recovery standards (SRSs). SRSs were used to correct for losses due to destruction of sugars during diluting acid hydrolysis. The sealed samples and SRSs were autoclaved for 1 h at 121 °C, and then slowly cooled to near room temperature. Acidic solutions were filtered and diluted to 100 times to analyze carbohydrate composition by high-performance anionic chromatography (Dionex ICS-3000, Dionex Corp., Sunnyvale, CA, USA).

The lignin fractionated by the acid hydrolysis includes acid soluble lignin (ASL) in the solution and acid insoluble lignin (AIL) in the insoluble residue. A portion of filtered acidic solution was diluted 2.5 times by deionized water using a pipette. The absorbance of this diluted solution at 240 nm (wavelength) was measured by a UV-Visible spectrophotometer (Lambda 25, Perkinelmer, Waltham, MA, USA) to determine the ASL. AIL was rinsed several times by deionized water, it was then oven-dried to determine its weight.

#### 3.4.2. FTIR Spectroscopy

The functional groups in the hydrolyzed residuals, MCC, and CNCs derived from them were determined by FTIR spectroscopy (Nicolet iS50 FT-IR, Thermo Nicolet, Madison, WI, USA) in the transmittance mode within a range of 3700–700 cm^−1^. The hydrolyzed residuals or MCC were mixed with potassium bromide (KBr) at a ratio of 1:100. The FTIR samples for the CNCs were prepared by adding KBr into the CNCs suspension at a solid ratio of CNCs:KBr = 1:100, which were then completely oven dried at 40 °C. All the samples were ground and then pressed into transparent pellets for FTIR analysis.

#### 3.4.3. Imaging

Morphologies of the hydrolyzed residuals (MMW and SPW), their corresponding celluloses, and CNCs were imaged by transmission electron microscopy (JEOL 1200 EX, Tokyo, Japan) operated at 100 kV. The solid samples were first dispersed in deionized water. A drop of sample suspensions was introduced onto a formvar- and carbon-coated copper grid and was stained with a drop of 1 wt % aqueous uranyl acetate. The microscopic samples stood in the ambient condition until water completely evaporated before TEM testing. Polarized optical microscopy (Olympus BX-51, Tokyo, Japan) was used to analyze the particle size of freeze-dried hydrolyzed residuals and celluloses by image analysis with imageJ software.

A simplified method to observe flow birefringence of the CNC suspension was developed. A regular light laptop screen (linearly polarized) or a light table with polarizing paper was used as a polarized light source. The CNC suspension was shaken in the front of the polarized light and photographed by a digital camera (Nikon D7000, Tokyo, Japan) covered with a polarized sun glass.

#### 3.4.4. Crystallinity

X-ray Diffraction (XRD) diffractograms of hydrolyzed residuals, cellulose, and CNCs were recorded using a Rigaku Miniflex 600 X-ray Diffractometer (Rigaku Co., Tokyo, Japan) with Ni-filtered Cu Kα radiation (λ = 0.15418 Å) at 45 kV, 40 mA, and a scanning step of 0.02° from 2θ = 10° to 40° at room temperature.

#### 3.4.5. Zeta Potential

The zeta potentials of 0.005 wt % CNC suspensions were determined using Malvern 3000 Zetasizer Nano ZS (Malvern Instruments, Malvern, UK) at a wavelength of 633 nm and a detecting angle of 173°. The samples were kept at a constant temperature of 25 °C for 2 min before registering the zeta potentials.

#### 3.4.6. Thermal Stability

Hydrolyzed residuals, cellulose, and CNCs were dried at 70 °C for 24 h before testing. Thermal decomposition was analyzed in terms of global mass loss by thermogravimetric analysis (TGA) (SDTQ600,TA Inc., New Castle, DE, USA) ramping from room temperature (30 °C) to 600 °C at a heating rate of 5, 10, 20, or 35 °C/min, respectively.

## 4. Conclusions

In this paper, chemical analysis and XRD evidenced the existence of cellulose and cellulose crystalline structure in bio-ethanol hydrolyzed residuals after enzymatic hydrolysis of mechanical or chemical pretreated softwood. These hydrolyzed residuals were purified and sulfuric acid was hydrolyzed to obtain the target CNC_MMW_ and CNC_SPW_. The resulted cellulose nanocrystals had a crystallinity of 73.7% and 74.0%, respectively, within a typical range of the crystallinity of CNCs from other sources but lower than 85.5% of CNC_MCC_. The particle size of CNC_MMW_ was smaller than CNC_MCC_’s, implying that the physical pretreatment and enzymatic hydrolysis in previous biorefinery cut the wood particles into smaller cellulose chains. However, CNCs from hydrolyzed residuals presented a better thermal stability due to the lower sulfate half esterification as indicated by combination of the zeta potentials and FTIR analysis. This study has demonstrated that bio-ethanol residuals are a source of crystalline cellulose to derive cellulose nanocrystals or potentially other cellulose nanoparticles. Therefore, with the purpose of utilizing the waste of biorefineries to increase biorefinery competiveness and improve the environmental benefits, co-production of bio-ethanol and bio-based materials should be encouraged in policy.

## Figures and Tables

**Table 1:** Chemical compositions of hydrolyzed residuals and microcrystalline cellulose (MCC).

Samples	Lignin Analysis		Carbohydrate Analysis			
	Acid-Insoluble Lignin	Acid-Soluble Lignin	Glucan	Mannan/Xylan	Arabinan	Galactan
	%	%	%	%	%	%
HR_MMW_	54.10	3.64	25.37	9.08	1.01	3.41
HR_SPW_	68.38	4.62	21.35	2.10	--	--
MCC	--	3.39	80.20	--	--	--

Note: -- refer to no value that is detected or obtained in the chemical analysis.

**Table 2:** Particle size of HR_MMW_, HR_SPW_, MCC, and their CNCs.

Samples	Length	Width	Samples	Length	Width	Samples	Length	Width
HR_MMW_	13 (5) μm	7 (4) μm	HR_SPW_	34 (19) μm	9 (7) μm	MCC	74 (42) μm	20 (10) μm
CNC_MMW_	202 (78) nm	16 (9) nm	CNC_SPW_	268 (118) nm	16 (8) nm	CNC_MCC_	270 (122) nm	17 (8) nm

Note: the values in brackets were standard deviations.

**Table 3:** Parameters of bioresiduals and their resulting cellulose nanocrystals.

Samples	Cr	*Z* Potential	*T*_onsetβ→0_	*T*_Pβ→0_	*E*_a_
	%	mV	°C	°C	kJ/mol
HR_MMW_	27.4	—	225.4	350.9	172.8
HR_SPW_	31.4	—	293.1	338.4	165.7
MCC	81.0	—	297.8	323.2	155.4
CNC_MMW_	73.7	−63.5	271.1	289.9	162.8
CNC_SPW_	74.0	−64.3	252.4	271.7	168.9
CNC_MCC_	85.5	−66.4	167.5	270.5	144.5

Note: the raw materials (HR_MMW_, HR_SPW_, MCC) could not be stably suspended in water to test *Z* potential.
